# Effects of a Simulated Acute Oil Spillage on Bacterial Communities from Arctic and Antarctic Marine Sediments

**DOI:** 10.3390/microorganisms7120632

**Published:** 2019-11-30

**Authors:** Carmen Rizzo, Roberta Malavenda, Berna Gerçe, Maria Papale, Christoph Syldatk, Rudolf Hausmann, Vivia Bruni, Luigi Michaud, Angelina Lo Giudice, Stefano Amalfitano

**Affiliations:** 1 Department of Chemical, Biological, Pharmaceutical and Environmental Sciences, University of Messina, 98166 Messina, Italy; carmen.rizzo@unime.it (C.R.); rmalavenda@unime.it (R.M.); 2 Institute of Process Engineering in Life Sciences, Section II: Technical Biology, Karlsruhe Institute of Technology (KIT), 76131 Karlsruhe, Germany; berna.gerce2@kit.edu (B.G.);; 3 Institute of Polar Sciences, National Research Council (CNR-ISP), 98122 Messina, Italy; mpapale@unime.it; 4 Department of Bioprocess Engineering, Institute of Food Science and Biotechnology, University of Hohenheim, 70599 Stuttgart, Germany; Rudolf.Hausmann@uni-hohenheim.de; 5 Water Research Institute, National Research Council (CNR-IRSA), 00015 Rome, Italy; amalfitano@irsa.cnr.it

**Keywords:** arctic, antarctic, sediment, microcosms, hydrocarbons, biodegradation, bioremediation

## Abstract

Background: The bacterial community responses to oil spill events are key elements to predict the fate of hydrocarbon pollution in receiving aquatic environments. In polar systems, cold temperatures and low irradiance levels can limit the effectiveness of contamination removal processes. In this study, the effects of a simulated acute oil spillage on bacterial communities from polar sediments were investigated, by assessing the role of hydrocarbon mixture, incubation time and source bacterial community in selecting oil-degrading bacterial phylotypes. Methods: The bacterial hydrocarbon degradation was evaluated by gas chromatography. Flow cytometric and fingerprinting profiles were used to assess the bacterial community dynamics over the experimental incubation time. Results: Direct responses to the simulated oil spill event were found from both Arctic and Antarctic settings, with recurrent bacterial community traits and diversity profiles, especially in crude oil enrichment. Along with the dominance of *Pseudomonas* spp., members of the well-known hydrocarbon degraders *Granulosicoccus* spp. and *Cycloclasticus* spp. were retrieved from both sediments. Conclusions: Our findings indicated that polar bacterial populations are able to respond to the detrimental effects of simulated hydrocarbon pollution, by developing into a more specialized active oil degrading community.

## 1. Introduction

Petroleum hydrocarbons are the main pollution source in polar ecosystems [1,2]. Although many petroleum products are used, stored, transported, and spilled in localized regions across the poles, the environmental contamination levels are hardly assessed or retrieved from country-specific documentations. In the Arctic, the risks of petroleum hydrocarbon pollution are increasingly because of the rising exploration activities, and a growing interest in developing the Northern Sea Route (NSR) as an alternative transportation route for oil and gas from Russia to Europe and other markets [3]. In the Antarctic, oil spills are known and registered events, but the spatial and temporal contamination patterns were not determined specifically, though wastes are managed under the common international environmental protocol (Madrid Protocol 1991).

Studies focused on Arctic and Antarctic seawater and ice samples showed that the introduction of hydrocarbons stimulated the selective growth of specialized hydrocarbon-degrading microorganisms [4,5]. They include mostly Proteobacteria members, mainly affiliated to *Pseudoalteromonas*, *Pseudomonas*, *Psychrobacter* spp. [6,7], *Marinobacter* [3,8], *Oleispira* [9], *Shewanella* [10], but also Bacteroidetes (e.g., *Cytophaga* spp.) and Actinobacteria (e.g., *Rhodococcus* spp.) [7,11,12,13,14]. The percentage of hydrocarbon-degrading bacteria were reported to increase up to ten folds, passing from 0.1 to 1% of the total heterotrophic bacteria in uncontaminated polar areas to 1–10% after an event of oil spill [12]. Moreover, consistent shift in the bacterial assemblage structure were detected immediately after the oil release into the environment [3]. In fact, multispecies microbial consortia can be more effective in oil degradation, through retaining a broader suite of enzymatic capacity than single microbial species, which grow on a limited range of carbon substrates [15].

The microbially driven mineralization processes can fundamentally contribute to hydrocarbon degradation and the natural attenuation of detrimental effects caused by oil spill [2]. The bacterial biodegradation potential is of utmost importance particularly in polar areas, since cold temperatures and light–dark cycles can limit the effectiveness of abiotic processes for contaminant removal [16].

Along with numerous field observations of microbial community responses to hydrocarbon contamination, the relative simplicity of experimental model systems is actively contributing to elucidate the bacterial potential for petroleum biodegradation in polar environments [17,18]. However, only few experimental studies analyzed the bacterial community patterns following oil spill of different composition [6], and the microbial responses to hydrocarbon contamination have not yet been evaluated concurrently in sediments from different polar regions.

This study was entailed to explore the effects of a hydrocarbon addition on the structural characteristics of bacterial communities in Arctic and Antarctic sediments. More specifically, we aimed at assessing (i) the aliphatic biodegradation patterns in sediments amended with different oil mixtures, (ii) the changes in bacterial community abundance (i.e., flow cytometry) and diversity profiles (i.e., Terminal Restriction Fragment Length Polymorphism analysis (T-RFLP)), and (iii) the occurrence of dominant bacterial species (i.e., Denaturing Gradient Gel Electrophoresis (DGGE)). By assuming a relevant impact of hydrocarbon amendments, we hypothesized direct responses from Arctic and Antarctic sediment microbial communities over the incubation time, with possible recurrent community traits that will occur because of the experimental stress factors.

## 2. Materials and Methods

### 2.1. Sampling Sites and Sediment Collection

Arctic samples were taken from the glacial open fjord Kongsfjorden, at the Research Village in Ny-Alesund (coordinates: 78°55′606′′N, 11°56′377′′E; Svalbard Archipelago, Arctic Norway) [7]. Antarctic samples were collected from the Byers Peninsula (Antarctic Specially Protected Area No. 126), at the mouth of the stream Petreles (coordinates: 62°40′09.9”S, 61°09′04”W; Livingston Island, Antarctica).

In each site of the north and south polar areas, during their respective summer periods in 2009, approximately 2 kg of sediment samples were collected between depths of 0 and 10 cm, together with seawater samples, by using sterile polycarbonate bottles, and stored in sterile polycarbonate bottles at 4 °C until processing. After arrival at the laboratory sediment samples were divided in subsamples used to set up microcosms. At the sampling days, seawater temperature and salinity were 7.9 °C and 22.8‰ and 6.0 °C and 30.0‰ in the Arctic and Antarctic sites, respectively.

### 2.2. Microcosm Set-Up

Hydrocarbon-enriched microcosms were set up in triplicates with Arctic and Antarctic samples by filling 250 mL glass beakers, covered with a sterile glass Petri dish cover to avoid external contamination during the incubation, with 150 g of sediment and 150 mL of filter-sterilized seawater from the same sampling site, as previously described [2]. Briefly, filter-sterilized crude oil (Arabian light) or commercial diesel oil (Q8 Italy) was added to each microcosm and mixed with a sterile glass stick (final concentration 1.5%, *v/v*). Hydrocarbon-free microcosms were used as control. All microcosms were incubated at 4 °C for 160 days. At established time intervals, microcosms were gently shaken and then left to settle. Finally, sediment subsamples (10 g) were aseptically collected with a sterile spoon from each microcosm for chemical and microbiological analyses.

### 2.3. Analysis of Residual Hydrocarbons in Arctic and Antarctic Sediments

The biodegradation efficiency was monitored by gas chromatographic analysis to achieve a qualitative/quantitative estimation of residual hydrocarbons in each microcosm. Residual hydrocarbons from sediment subsamples (100 g) at the experiment beginning (0 days), after 90 days of incubation and end (160 days), were extracted using methyl-tertiary-butyl-ether:hexane (20:80), a modified version than the reference standard EPA (SW-846 3550B-Ultrasonic Extraction). Time 0 samples were used as control for abiotic losses. The ultrasound-based process guaranteed a strong adhesion of the sample matrix to the solvent extraction. The entire sample was transferred in a dark glass bottle, using sodium sulfate (5 g) as drying agent. The liquid layer of solvent was poured through a funnel built with filter (Whatman N° 2) Na_2_SO_4_ in a 250 mL pre-equilibrate evaporation flask. The extract was concentrated to a small volume by evaporation under reduced pressure in a rotavapor, and 1 mL was pipetted into glass vials with Teflon cap and refrigerated until analysis by gas chromatography. Immediately before the extraction, butylhydroxytoluene (BHT) was added as a surrogate standard. The standards used to monitor the biodegradation of crude oil and diesel oil were a mixture containing from C7 to C30 fractions and a mixture containing fractions from C13 to C18, respectively. The characterization of petroleum product was made after solvent extraction methods followed by gas chromatography analysis with a Flame Ionization Detector (FID). A gas chromatograph GC Series 17-A (Shimadzu Ltd., Kagoshima, Japan) equipped with Electron Capture detector (ECD) and FID and AOC-5000 autosampler (CombiPAL) was used for analysis. Analyses were conducted using Petrocol™ column (Supelco, St. Louis, MO, USA): 100 m long with inner diameter of 0.25 mm and the carrier material with diameter of 0.5 µm. The temperature of the injector and detector were set at 280 °C. We used a temperature program, which provides a better separation of various components with different boiling temperatures. The initial temperature of the gas chromatograph oven temperature control was maintained at 35 °C for ~15 min, and then the temperature was increased by 2 °C per minute to a temperature of 200 °C. The last step in temperature was set at 300 °C with an increase in temperature of 10 °C per minute and maintained for 2.5 min for a total analysis in 110 min. Helium was used as carrier gas at about 20 cm s^−1^, nitrogen as make-up gas at 30 mL min^−1^. This method allowed the separation of all the components of the matrix with particular reference to petroleum. The retention time was determinate by applying defined standard containing a mixture of compounds from C7 to C30 (Supelco) at a concentration of 1 mg mL^−1^ for each component in hexane solvent. The calibration curve for the quantitative analysis was conducted using five standards and increasing the concentration by using the gas chromatographic procedure management software provided by Shimadzu Class VP 7 Ltd. All lines obtained had a correlation coefficient greater than 0.95. Control of retention times was also conducted with the calculation of linear retention indices (LRI).

### 2.4. Microbial Community Characterization

#### 2.4.1. Flow Cytometry

Total cell counts were assessed at regular time intervals for both treatments (i.e., 0, 30, 60, 80, 90, 120, and 160 days of incubation). Prokaryotic cells were detached from sediments by mixing sample aliquots (1 g) with phosphate buffered saline (PBS: 120 mM NaCl, 2.7 mM KCl in 10 mM phosphate buffer, pH 7.6; 9 mL), sodium pyrophosphate (0.1%), and tween 80 (10 µL). The mixture was fixed with formalin solution (final concentration 2%, *v/v*), shaken vigorously for 20 min, and stored at 4 °C until processing [19]. The total prokaryotic abundance was estimated by using the Flow Cytometer A-50 (Apogee Flow System, Hertfordshire, England), as described in Amalfitano et al. [20]. The Apogee Histogram Software v89.0, Apogee Flow System, Hertfordshire, England) was used for data elaboration, which were expressed as cells per gram of wet sediment (cells g^−1^).

#### 2.4.2. DNA Extraction and Fingerprinting Analyses

The total DNA was extracted from 500 mg of starting material using a bead beating kit (FastDNA^®^ SPIN Kit for Soil, Qbiogene, Heidelberg, Germany) following the manufacturer’s instructions. Two fingerprinting approaches were used for microbial community characterization on selected sampling times: at the microcosm set up (0 days), after 30 days, 90 days, and 160 days of incubation. As hydrocarbons degradation and emulsification rate appeared optically conspicuous in Arctic microcosms, an additional intermediate sampling was performed after 60 days of incubation.

The Terminal Restriction Fragment Length Polymorphism analysis (T-RFLP) was used to evaluate the diversity profiles of bacterial communities over time. The amplification of genes coding for 16S rRNA was carried out by PCR, with reaction mixture and conditions as previously described [12]. PCR reactions have been performed with a PTC-100 Peltier Thermal Cycler (MJ Research Celbio). Amplification products of three parallel PCR reactions were combined and purified with the Wizard SV Gel and PCR Clean-Up system (Promega, Durham, NC, USA) prior to perform T-RFLP analysis, as described in the following sections. 

Approximately 300 ng of amplified 16S rRNA genes from each DNA sample were digested in duplicate with 10U of *Alu*I restriction enzyme (Fermentas). Denaturation of reaction mixtures were carried out by heating at 95 °C for 3 min, and then incubated at 0 °C for 5 min. Samples were then sent to an outsourcing sequencing service (BMR-Genomics, Italy). The reaction outputs (electropherograms) were processed using methods as described by Luna et al. [21]. The “Abundance percentage” (Ap) of each T-RF was calculated as indicated by Lokow et al. [22]. The size of each T-RF was estimated in reference to an internal standard. All subsequent processing phases of T-RFLP data have been performed as suggested by Smith et al. [23] and Baldi et al. [24], to characterize the microbial community diversity patterns. After enzymatic digestion of PCR amplicons, each T-RF can be defined as an operational taxonomic unit (OTU) within a community [25]. Statistical analysis have been computed by considering the number of peaks as indicator of the species number (phylotype/genotype richness) and the band intensity peak height as the relative abundance of each bacterial species [26].

Denaturing Gradient Gel Electrophoresis (DGGE) was used to promptly identify the dominant bacterial species occurring in contaminated Arctic and Antarctic sediments over the experimental incubation time. The variable V3 region of the 16S rDNA of bacteria was amplified by using universal primers (27F, 5′-AGA GTT TGA TC(AC) TGG CTC AG-3′ with GC-clamp in position 5′-CGC CCG CCG CGC CCC GCG CCC GTC CCG CCG CCC CCG CCC G-5′ spanning *Escherichia coli* position 8-27; 518R, 5′-ATT ACC GCG GCT GCT GG-3′ spanning *E.coli* position 518-534; - Biomers.net GmbH - Ulm, Germany). PCR was performed using a thermocycler (Mastercycler, GeneAmp PCR-System 9700, Applied Biosystems, Foster City, CA 94404, USA), as described by Gerçe et al. [27], and the same reference was used also for all DGGE conditions. A presence/absence matrix of bands was constructed for DGGE gel analysis (Alpha Imager 2.200; Biozym Scientific GmbH, Oldendorf, Germany).

Selected DGGE bands were carefully cut out under UV lamp with sterile scalpels, eluted overnight in 50 μL water at 4 °C (DNA-free PCR water, Molzym GmbH and Co. KG, Bremen, Germany), and PCR re-amplified using the DNA diffuses into the water as starting material in a PCR. PCR conditions were the same as described before with some modifications [27]. The NCBI GenBank database (http://www.ncbi.nlm.nih.gov) was used for comparing the sequences to 16S ribosomal RNA (rRNA) gene sequences by using the Basic Local Alignment Search Tool (BLASTN) algorithm [28].

### 2.5. Statistical Analyses

To examine the differences in bacterial community profiles over time, fingerprint data were analyzed by presence⁄absence and relative abundance-based matrices using semimetric Bray–Curtis distance measures. Bray–Curtis similarities were calculated on both T-RFLP and DGGE data and used for Cluster analysis and visualized in a lower dimensional space by applying non-metric multidimensional scaling (nMDS). Statistical differences between bacterial community structures in the multivariate dataset by carrying out the Analysis of SIMilarities (ANOSIM). All calculation were carried out by the software Primer 6 (v. 6β R6, PRIMER-E Ltd., UK). Two-way ANOVA was performed to establish the effect of incubation time, treatment (diesel oil and crude oil), and sediment origin (Arctic and Antarctic) on the bacterial community diversity.

## 3. Results

### 3.1. Residual Hydrocarbons in Arctic and Antarctic Microcosms

**Figure 1:**
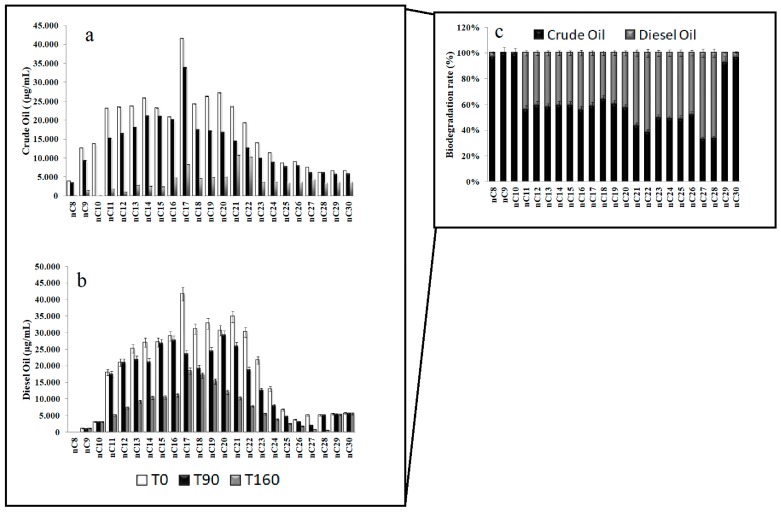
Residual hydrocarbons in Arctic microcosms enriched with crude oil (**a**) and diesel oil (**b**) over the incubation time (0, 90, and 160 days of incubation). Overall biodegradation of hydrocarbon mixtures (**c**).

The Arctic community was more efficient in the degradation of small chain hydrocarbons (except C-10) in the experiment setting with addition of crude oil, as it was demonstrated by the removal of hydrocarbons with C-10 and C-12 (Figure 1a), with biodegradation rates of 99% and 95% (Figure 1c). The fractions between C-16 and C-30 were optimally degraded but with a minor extent, except for the hydrocarbons with C-21 and C-22 chains, which were almost present also at the end of the experiment. Sediments in the microcosm enriched with diesel oil showed a very efficient community in the biodegradation of longer chain hydrocarbons (from C-21 to C-25), reaching up to 75% of their removal (Figure 1b). The Figure 1c shows more clearly the overall biodegradation rate for crude oil and diesel oil during the experiment.

**Figure 2:**
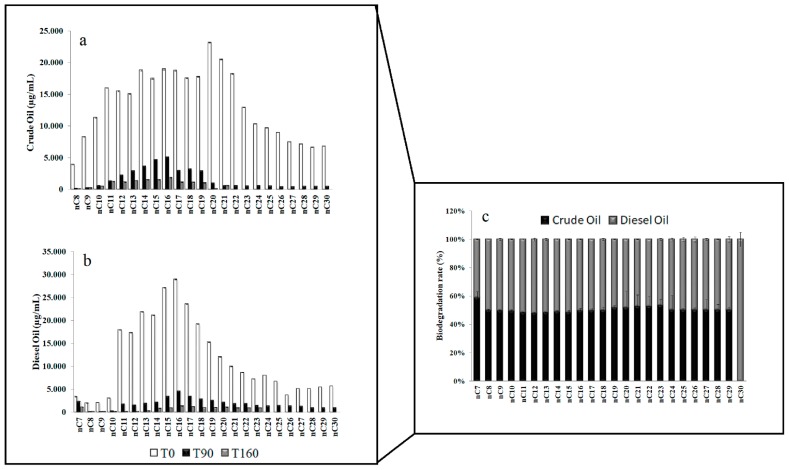
Residual hydrocarbons in Antarctic microcosms enriched with crude oil (**a**) and diesel oil (**b**) over the incubation time (0, 90, and 160 days of incubation). Overall biodegradation of hydrocarbon mixtures (**c**).

A relevant reduction of long chain hydrocarbons was found in Antarctic sediments (Figure 2a–c). In particular, at the end of the experiment the hydrocarbon chains from C-20 to C-30 in crude oil (Figure 2a), and chains from C-23 to C-30 in diesel oil (Figure 2b) were totally removed.

### 3.2. Patterns of Microbial Cell Abundance in Arctic and Antarctic Contaminated Sediments

In Arctic sediments, the microbial abundance varied between 0.2 × 10^6^ cells g^−1^ and 3.1 × 10^6^ cells g^−1^, with values increasing over time only in presence of crude oil. In Antarctic sediments, cell abundance reached values up to 14.4 and 9.1 × 10^6^ cells g^−1^ (in crude oil and diesel, respectively), and likely decreased along the incubation time.

**Figure 3:**
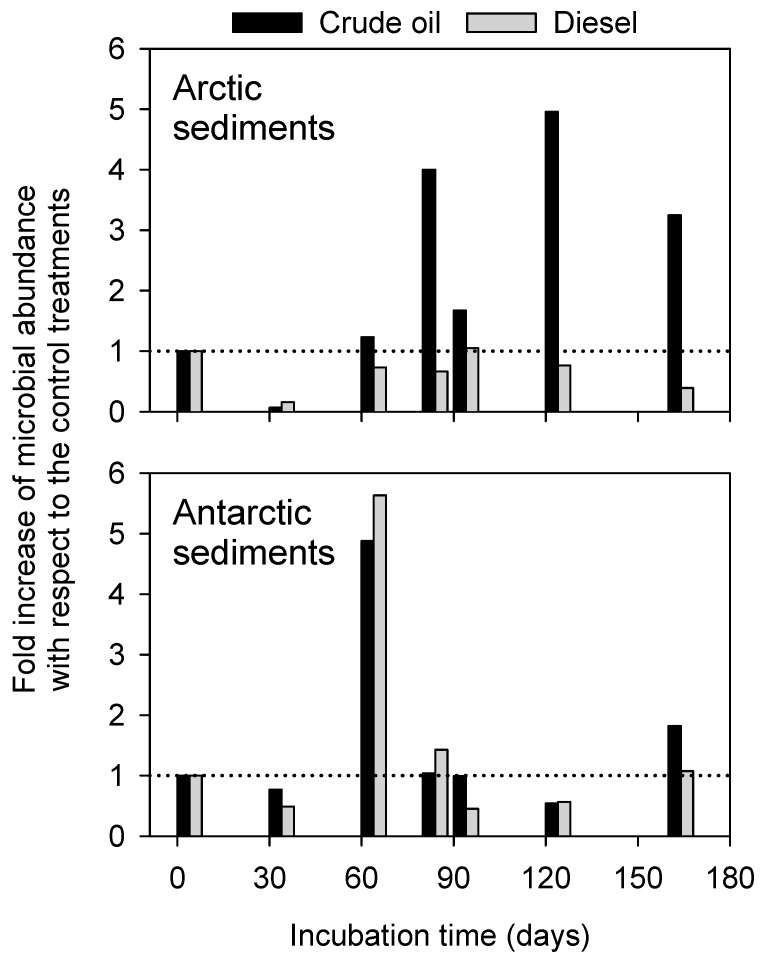
Patterns of the total prokaryotic cell counts as assessed by flow cytometry in Arctic and Antarctic microcosms. Data are presented as fold increase of microbial abundance in contaminated sediments with respect to the control treatments.

The fold increase of microbial abundance with respect to the relative control treatments (i.e., treatment-to-control ratio) was plotted to show the effects of oil contamination over time (Figure 3). As indicated by values below 1, a detrimental impact by oil contamination was evident at day 30 in all contaminated sediments, also reflecting the highest abundance values found in both control sediments (i.e., 79.1 × 10^6^ cells g^−1^ and 18.7 × 10^6^ cells g^−1^ in Arctic and Antarctic sediments, respectively). In Arctic sediments, the detrimental effects of diesel contamination were evident throughout the entire incubation period. From day 60 in sediments with crude oil, fold increase values exceeded 1, thus indicating a possible hydrocarbon-stimulated increase of microbial abundance. A similar pattern was found in Antarctic sediments contaminated by diesel oil.

### 3.3. Community Diversity Profiles in Contaminated Sediments over the Incubation Time

**Figure 4:**
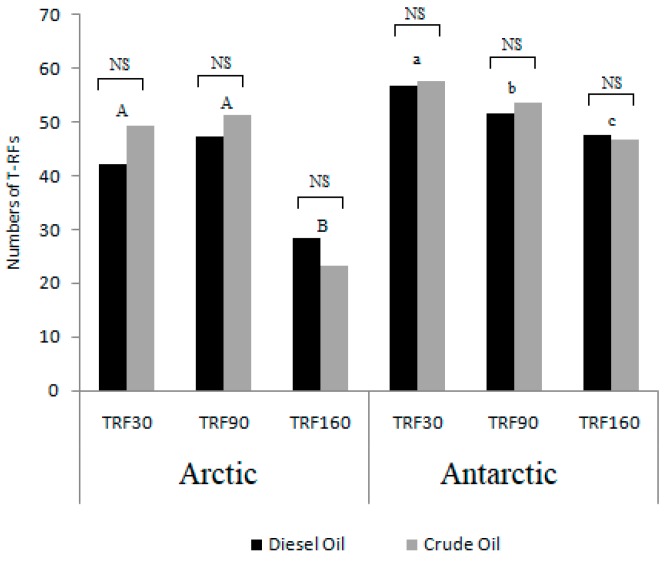
The bacterial richness expressed as the number of T-RFs (T-RFLP) detected in the microcosms samples from two experimental treatments (crude oil and diesel oil) in three sampling times (TRF30: 30 days of incubation; TRF60: 60 days of incubation; TRF160: 160 days of incubation). The different simple and capital letters denote statistically significant differences among each sampling times in Arctic and microcosms; NS—not significant different (ANOVA, *p* = 0.05).

A number of t-RFs ranging from 23 to 63 in the bacterial community profiles were obtained from T-RFLP analysis. Most t-RFs were identified at the Arctic sediments after 0 days of incubation in crude oil treatment, and in the Antarctic sediments after 30 days of incubation both in diesel oil and crude oil treatment. The minimum number was detected in Arctic sediments at the end of the experiments in both the microcosm settings. No statistically significant differences were found between treatments (i.e., diesel oil or crude oil at each time), while significant differences occurred between sampling times (Figure 4). In detail, for the Arctic microcosm the t-RFs after 160 days of incubation were significantly different from those detected after 30 and 90 days of incubation. In the case of Antarctic microcosm, significant differences occurred between all sampling times, namely 30, 90 and 160 days.

As calculated from the T-RFLP data, the highest genetic diversity and evenness were detected in Arctic sediments with crude oil after 90 days of incubation and Antarctic sediments with diesel oil after 30 days of incubation (Figure S1). In both Arctic and Antarctic microcosms, richness globally decreased over time after the addition of hydrocarbons.

In Arctic sediments, the T-RFLP analysis revealed that the bacterial community structure had differences over time in the treatment with crude oil, as demonstrated by the low Bray–Curtis similarity. The sediments enriched with crude oil exhibited the higher dissimilarity rate over time with 32.4% of similarity between the different time samplings. The community appeared more similar over time in the microcosm supplemented with diesel oil and in the control setting, showing a similarity of 42.3% and 56.9% between sampling times, respectively. Similarity percentage of 35.0% (ANOSIM Global *R* > 0.4; *P* > 0.8) was detected when crude oil and diesel oil settings were compared, while a similarity of 47.1% (ANOSIM Global *R* > 0.2; *P* > 0.01) was recorded in case of comparison with the control experiment (Figure S2a).

Similarly, the T-RFLP analysis suggest an influence of hydrocarbon introduction on the Antarctic bacterial community structure (ANOSIM Global *R* > 0.4; *P* > 0.08). Results obtained for the Antarctic sediments enriched with crude oil showed an average similarity of 61.8% between sample aliquots collected during the time course experiment, and 59.4% (ANOSIM Global *R* > 0.7; *P* > 0.2) in comparison with the control. Differently, the bacterial community of microcosm supplemented with diesel oil showed a similarity of 81.0% between aliquots collected at different sampling times, and a value of 68.7% (ANOSIM Global *R* > 0.6; *P* > 0.5) compared with the control (Figure S2b).

### 3.4. Occurrence of Dominant Bacterial Species across the Experimental Conditions

The DGGE gel obtained from the Arctic sediments revealed the presence of a total of 30 dominant phylotypes and a banding pattern of 11 to 18 bands (Figure S3). A total of 77 bands were excised from gel and analyzed for taxonomical affiliation by 16S rRNA gene sequencing. Among them, 30 were considered as phylotype representatives (Table 1 and Figure S3). The phylogenetic analysis of partial 16S rRNA gene sequences from selected DGGE bands revealed that Arctic bacteria were related to the Proteobacteria (Alpha-, Beta-, Delta-, and Gammaproteobacteria), Actinobacteria, Firmicutes, and CF group of Bacteroidetes. *Pseudomonas frederiksbergensis* (DGGE_band_114), *Pseudomonas costantinii* (DGGE_band_123), and *Magnetospirillum gryphiswaldense* (DGGE_band_131) were observed only in the presence of diesel oil (from 30 to 160 days of incubation, and from 60 to 160 days of incubation, respectively), whereas *Magnetospirillum magnetotacticum* (DGGE_band_95) only in the presence of crude oil (from 30 to 90 days of incubation). A number of sequences were retrieved both in presence of crude oil and diesel oil at almost all sampling times, and shared with the control setting: *Kofleria flava* (DGGE_band_62), *Granulosicoccus antarcticus* (DGGE_band_66), *Marinobacter antarcticus* (DGGE_band_69), *Rhodoferax fermentans* (DGGE_band_70), *Pibocella ponti* (DGGE_band_115), *Pseudomonas congelans* (DGGE_band_121), and *Pseudomonas sabulinigri* (DGGE_band_129).

**Figure 5:**
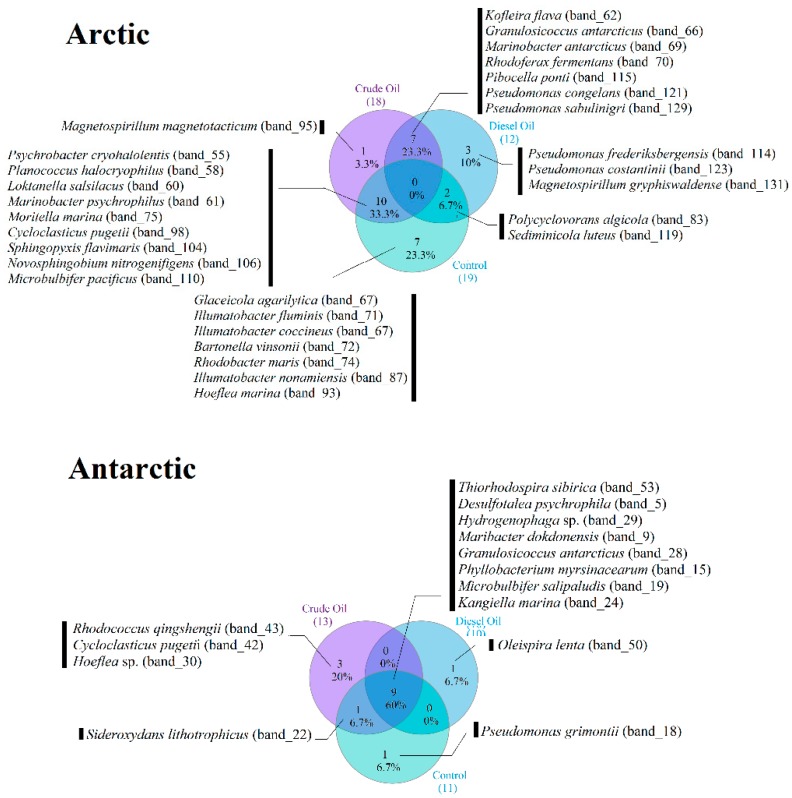
Venn diagrams showing phylotypes distribution in Arctic and Antarctic microcosms detected by denaturing gradient gel electrophoresis (DGGE) analysis.

Other phylotypes were shared between the control experiment and the crude oil treatment or diesel oil treatment. The occurrence of sequences observed only in the absence of hydrocarbons was also verified (Figure 5).

The DGGE gel obtained from the Antarctic sediments revealed the presence of 23 dominant phylotypes and a banding pattern of 12 to 14 bands (Figure S4). A total of 53 bands were excised from DGGE gels and characterized by 16S rRNA gene sequence analysis, resulting in 15 phylotype representatives that gave sequencing results (Table 1 and Figure S4). The phylogenetic analysis of partial 16S rRNA gene sequences from selected DGGE bands revealed that bacteria in the Antarctic microcosm were related to the Proteobacteria (Alpha-, Beta-, Gamma-, and Deltaproteobacteria), Actinobacteria, and CF group of Bacteroidetes. A shift in diversity and the emergence of a small number of dominant bands compared to the corresponding control treatment were observed. The crude oil and diesel oil addition during incubation resulted in a shift towards the appearance or disappearance of specialized hydrocarbon degraders. For example, *Oleispira lenta* (DGGE_band_50) sequences were retrieved only in Antarctic microcosm supplemented with diesel oil after 90 days of incubation. The same was true for *Rhodococcus qingshengii* (DGGE_band_43) in Antarctic microcosms with crude oil, even if it was observed also after 160 days of incubation. *Cycloclasticus pugetii* (DGGE_band_42) became visible only at the end of the experiment with addition of crude oil (160 days), while *Hoeflea* sp. (DGGE_band_30) was observed only in the same treatment after 30 days, but then it disappeared.

As for Arctic sediments, a number of sequences were retrieved in both hydrocarbon-enriched microcosms at all sampling times, and shared with the control. The betaproteobacterium *Sideroxydans lithotrophicus* (DGGE_band_22) co-occurred in control and crude oil treatments, but it was absent in the diesel oil treatment. *Marinimicrobium agarilyticum* (DGGE_band_18) appeared in the control only at the end of the incubation. Finally, sequences from *Pseudomonas grimontii* (DGGE_band_51) were retrieved from all microcosms (including the control) after 160 days. *Salinibacterium amurskyense* (DGGE_band_8), visible at 0 days of incubation, disappeared after hydrocarbon addition.

**Figure 6:**
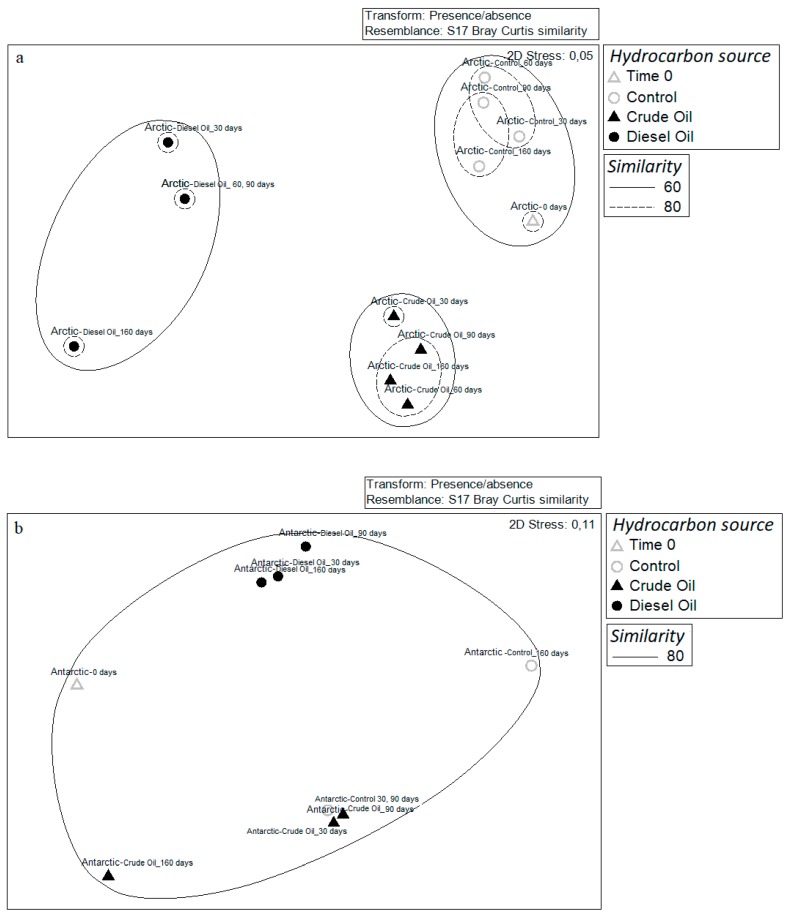
Non-metric multidimensional scaling (nMDS) computed on Bray–Curtis similarities calculated presence/absence matrix obtained from DGGE analysis for (**a**) Arctic and (**b**) Antarctic microcosms.

**Figure 7:**
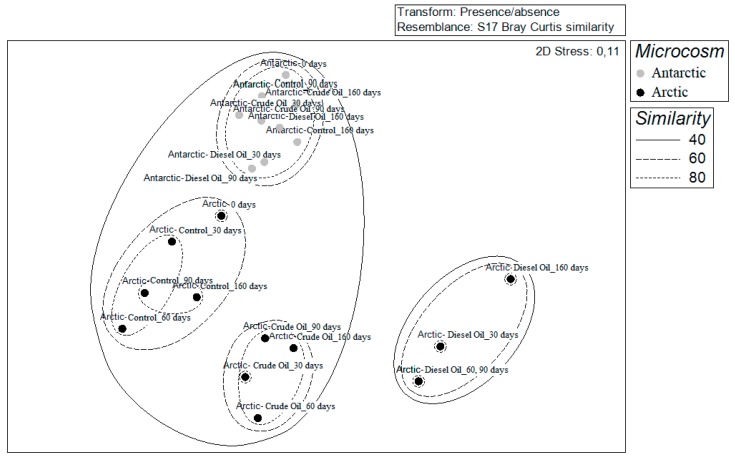
Non-metric multidimensional scaling (nMDS) computed on Bray–Curtis similarities calculated from DGGE analysis results, plotted by clustering data according to sediment origin.

Comparison of the Bray–Curtis dissimilarity matrices showed a similarity within Antarctic microcosms communities (both crude oil and diesel oil treatments) of 91% and 98%, respectively. Antarctic-crude oil and Antarctic-diesel oil communities showed similar values of similarity (88.5% and 88.7%, respectively) with the control experiment (Figure 6a). With regard to the Arctic sediments, this analysis revealed that the communities grouped differently in dependence of the hydrocarbon enrichment. The similarity within the different communities over the set time was always higher than 80%, but it drastically decrease when compared to the community from the control microcosm with 57 and 47% of similarity to Arctic-crude oil and Arctic-diesel oil, respectively (ANOSIM Global R = 0.9; *P* < 0.03) (Figure 6b). The nMDS analysis computed on DGGE matrix results clearly showed the different clustering of polar communities in response to hydrocarbon addition, by highlighting the occurrence of a separate cluster grouping all samples from Antarctic microcosms, whereas Arctic microcosm samples formed three main clusters, one for crude oil treatment samples, a second for diesel oil treatment samples, and the last one for control samples (Figure 7).

## 4. Discussion

This study aimed to investigate the effects of an acute oil spillage on microbial communities from polar sediments, also verifying the possible analogies/differences in the occurring possible specific responses related to the community origin.

A different response was highlighted in the biodegradation processes on the two hydrocarbon mixtures. Generally, the trend in hydrocarbon degradation involves a faster removal of short and medium chain length alkanes than the long chain length alkanes. Despite this, different patterns have been reported. For example, Mason et al. [29] detected a faster degradation of dodecane than phenanthrene and toluene, whereas Yergeau at al. [30] and Bacosa et al. [31] reported faster polycyclic aromatic hydrocarbon degradation within 30 days of incubation. In our case, the gas chromatographic analysis on residual aliphatic hydrocarbons at the end of incubation showed that the Arctic community was able to better utilize hydrocarbons from C13 to C30 in crude oil. On the other hand, the Antarctic community was more efficient (up to 100% per single compound removed) in the biodegradation of longer chain hydrocarbons (crude oil from C7 to C19; diesel oil from C7 to C22). This finding might rely on the different composition of the source microbial community that naturally occurred at the diverse levels of pollution reported from the sampling Arctic and Antarctic areas, as it was confirmed by the differences encountered between DDGE results. However, the bacterial community dynamics showed similar patterns in the two microcosm settings in terms of bacterial abundance. The hydrocarbon contamination was likely limiting the microbial cell growth at the first month from the simulated oil spill event. A possible growth-stimulating effect was found afterwards, with higher cell abundance values found in contaminated sediments than in the control treatment. The first biodegradative processes, and the consequent appearance of hydrocarbon intermediates from reactions, could in fact contribute to the development of a bacterial community specialized in the degradation of hydrocarbons, with increase of cell abundance. The sediment bacterial communities can develop into active oil degrading consortia after oil contamination, despite hydrocarbon-degrading bacteria are recurrently reported even in uncontaminated areas [13]. Obligate hydrocarbonoclastic bacteria may occur immediately after the release of oil into the environment, by becoming dominant component of the microbial community [32,33,34].

Although an increase in abundance of the cultivable bacterial fraction is also reported in response to hydrocarbons, the bacterial diversity can dramatically decrease [35]. Our findings pointed to higher specialized communities at the end of the experiment in terms of diversity profiles.

Despite recent advances in genomics and sequencing technologies, molecular approaches have been scantly applied to similar experimental study about effects of a simulated oil spill event on polar bacterial communities. Here, T-RFLP analysis was chosen as a semiquantitative and highly reproducible method [36,37], and it was a useful tool for investigating the species diversity and evenness, despite the phylogenetic characterization is limited to the observation and comparison of terminal restriction fragment (t-RF) [38,39]. This technique was coupled with DGGE, which is less sensitive than T-RFLP [40,41,42], but can better reflect the community composition, by evidencing potential dominant phylotypes.

Further similarities between Arctic and Antarctic communities were underlined by the changes in diversity profiles and by the appearance of specialized phylotypes over time. Both communities were affected by the hydrocarbon addition if compared with the control microcosm, and changes in bacterial diversity were also detected also in the time course between the different sampling times. The harsher crude oil effect on diversity profiles represented a common point in the two polar microcosms, as proven also by the similarity average calculated by the Bray–Curtis analysis, in comparison with the control and the diesel oil treatment, thus suggesting a more disturbing effect of crude oil on microcosms. Indeed, the similarity in crude oil treatment was lower, and this was particularly true for Arctic microcosm, as demonstrated by the low Bray–Curtis similarity (32.4% and 61.9% for Arctic and Antarctic, respectively).

The appearance of bacterial species specialized in the hydrocarbon degradation supports the detrimental effect of crude oil and diesel oil on microbial communities structure. Some species appeared in relation to the addition of hydrocarbon substrates, not only related to aliphatic hydrocarbon isoforms. For example, *Oleispira lenta* was found exclusively within the diesel oil enriched Antarctic community, whereas *Rhodococcus qingshengii* and *Cycloclasticus pugetii* (well-known degraders of polycyclic aromatic hydrocarbons) were both retrieved only in crude oil-enriched microcosm. Rhodococci are known as dominant alkane degraders in polar environment [35,43,44], despite a less pronounced cold adaptation than, for example, *Oleispira* spp. strains, obligate hydrocarbonoclastic [45,46]. Previous investigations in microcosm at low temperatures highlighted the bloom of organisms related to *Oleispira* sp. oil-degrading microbial communities [47,48]. Conversely, *Pseudomonas grimontii* sequences were obtained from both crude oil and diesel oil enriched Antarctic microcosms. The appearance of such bacterial sequences suggests a great versatility of the genus members, able to use as carbon source hydrocarbon fractions present in both substrates. For many years, members of the genera *Pseudomonas* and *Cycloclasticus* have been investigated for their hydrocarbon degradative properties, and were found in cold environments [2,49,50]. The different time of appearance confirmed that the several members of hydrocarbon-degrading community tend to occupy distinct trophic niches, and generally the aliphatic-degraders bloom first, followed by degraders of more complex and less bioavailable hydrocarbons [51].

Similarly to the experimental conditions here observed, observations in various marine habitats have shown increases in the occurrence of Gammaproteobacteria after oil contamination [15], even if the Alphaproteobacteria component resulted also well represented in the Arctic microcosm. The changes observed in the Arctic community in terms of taxonomic composition highlighted differences between the control and the contaminated microcosms. The species *Magnetospirillum magnetotacticum*, *Sediminicola luteus*, *Microbulbifer pacificus*, *Sphingopyxis flavimaris*, and *Thiobacillus thioparus* were found only in sediments with crude oil, whereas *Cycloclasticus pugetii*, *Novosphingobium nitrogenifigens*, *Pibocella ponti*, *Magnetospirillum gryphiswaldense*, and *Pseudomonas* spp. appeared in sediments with diesel oil. These taxa are known as aromatic hydrocarbon biodegraders [52,53]. Interestingly, the species of *Microbulbifer*, *Thiobacillus*, and *Novosphingobium* were never reported in cold environment, and only some references are available for *Magnetospirillum*, *Sphingopyxis*, and *Sediminicola* [4,54,55].

## 5. Conclusions

In this study, a simulated acute oil spillage had direct effects on the microbial community structure from polar sediments, showing notable analogies regardless the Arctic and Antarctic community origin. Both communities showed a similar bacterial abundance variation, with the occurrence of specialized phylotypes. Major changes in bacterial diversity were observed in relation to the incubation time rather than to hydrocarbon content and sediment origin.

The study outcomes provided an environmental relevant contribute by highlighting the selection of microbial consortia with higher potential in case of oil spills in polar areas, thus contributing to pave the way for novel biotechnological applications of sediment polar communities in bioremediation processes.

## Figures and Tables

**Table 1:** 16S rRNA gene sequence affiliation of selected DGGE bands to their closest phylogenetic neighbors. Sequences that were found in both microcosms are in bold.

Site	Phylum or Class	DGGE Bands	Next relative by Genbank Alignment (Accession Number, Microorganism)	Hom (%)
**MicroBy**	*Alphaproteobacteria*	30	KC160704, *Hoeflea* sp. SS10.8	86
		15	NR_113874, *Phyllobacterium myrsinacearum* strain NBRC100019	94
	*Betaproteobacteria*	29	JQ799976, *Hydrogenophaga* sp. FS13-2	99
		22	NR_074731, *Sideroxydans lithotrophicus* strain ES-1	82
	*Gammaproteobacteria*	**42**	**NR_025955**, *Cycloclasticus pugetii***PS-1**	**93**
		**28**	**NR_044255**; *Granulosicoccus antarcticus***IMCC3135**	**99**
		24	NR_109475, *Kangiella marina* strain KM1	90
		18	NR_043223, *Marinimicrobium agarilyticum* strain M18	93
		19	NR_025232, *Microbulbifer salipaludis* strain SM-1	97
		50	NR_108293, *Oleispira lenta* strain DFH11	99
		51	NR_025102, *Pseudomonas grimontii* CFML 97-514	99
		53	NR_028867, *Thiorhodospira sibirica* strain A12	95
	*Deltaproteobacteria*	5	NR_028729, *Desulfotalea psychrophila* LSv54	93
	*Bacteroidetes*	9	NR_043294, *Maribacter dokdonensis* strain DSW-8	89
	*Actinobacteria*	43	KT962173, *Rhodococcus qingshengii* strain CN-S1	100
		8	KF306368, *Salinibacterium amurskyense* strain y182	95
**MicroSval**	*Alphaproteobacteria*	74	NR_104902, *Bartonella vinsonii* subsp. *Arupensis* strain OK 94-513	93
		93	NR_043007, *Hoeflea marina* strain LMG 128	93
		60	NR_025539, *Loktanella salsilacus* strain R-8904	100
		95	NR_026381, *Magnetospirillum magnetotacticum* DSM 3856	89
		131	NR_121771, *Magnetospirillum gryphiswaldense* strain MSR-1	86
		106	NR_043857, *Novosphingobium nitrogenifigens* DSM 19370 Y88	95
		87	NR_042629, *Rhodobacter maris* strain JA276	88
		104	NR_025814, *Sphingopyxis flavimaris* strain SW-151	99
	*Betaproteobacteria*	70	NR_104835, *Rhodoferax antarcticus* strain ANT.BR	92
		111	NR_117864, *Thiobacillus thioparus* strain THI 111	95
	*Deltaproteobacteria*	62	NR_041981, *Kofleria flava* strain Pl vt1	92
	*Gammaproteobacteria*	**98**	**NR_025955**, *Cycloclasticus pugetii***PS-1**	**97**
		67	NR_043956, *Glaciecola agarilytica* strain NO2	94
		**66**	**NR_044255**, *Granulosicoccus antarcticus***IMCC3135**	**99**
		83	NR_116560, *Polycyclovorans algicola* strain TG408	99
		61	NR_043513, *Marinobacter psychrophilus* strain BSi20041	86
		69	NR_108299, *Marinobacter antarcticus* strain ZS2-30	92
		110	NR_11592, *Microbulbifer pacificus* strain SPO729	95
		75	NR_040842, *Moritella marina* strain ATCC15381	92
		121	NR_028985, *Pseudomonas congelans* strain P 538/23	99
		123	NR_025164, *Pseudomonas costantinii* strain CFBP 5705	99
		114	NR_028906, *Pseudomonas frederiksbergensis* strain JAJ28	100
		129	NR_044415, *Pseudomonas sabulinigri* strain J64	99
		55	NR_043079, *Psychrobacter cryohalolentis* K5	100
	*Bacteroidetes*	119	NR_041301, *Sediminicola luteus* strain CNI-3	99
		115	NR_025821, *Pibocella ponti* strain KMM 6031	93
	*Actinobacteria*	72	NR_112714, *Ilumatobacter coccineus* strain YM16-304	92
		71	NR_041633, *1Ilumatobacter fluminis* strain YM22-133	93
		88	NR_112713, *Ilumatobacter nonamiensis* strain YM16-303	95
	*Firmicutes*	58	NR_118149, *Planococcus halocryophilus* strain	100

## Supplementary Materials

Supplementary materials can be found at https://www.mdpi.com/2076-2607/7/12/632/s1.

## References

[1] Cripps G.C., Shears J. (1997). The fate in the marine environment of a minor diesel fuel spill from an Antarctic research station. Environ. Monit. Assess..

[2] Malavenda R., Rizzo C., Michaud L., Gerce B., Bruni V., Syldatk C., Hausmann R., Lo Giudice A. (2015). Biosurfactant production by Arctic and Antarctic bacteria growing on hydrocarbons. Polar Biol..

[3] Gerdes B., Brinkmeyer R., Dieckmann G., Helmke E. (2005). Influence of crude oil on changes of bacterial community in Arctic sea-ice. FEMS Microbiol. Ecol..

[4] Delille D., Pelletier E. (2002). Natural attenuation of diesel-oil contamination in a subantarctic soil (Crozet Island). Polar Biol..

[5] Powell S.M., Snapeb I., Bowmana J.P., Thompsona B., Starkb J., McCammon S.A., Riddleb M.J. (2005). A comparison of the short term effects of diesel fuel and lubricant oils on Antarctic benthic microbial communities. J. Exp. Mar. Biol. Ecol..

[6] Deppe U., Richnow H.H., Michaelis W., Antranikian G. (2005). Degradation of crude oil by an Arctic microbial consortium. Extremophiles.

[7] Conte A., Papale M., Amalfitano S., Mikkonen A., Rizzo C., De Domenico E., Michaud L., Lo Giudice A. (2018). Bacterial community structure along the subtidal sandy sediment belt of a high Arctic fjord (Kongsfjorden, Svalbard Islands). Sci. Total Environ..

[8] Lo Giudice A., Bruni V., De Domenico M., Michaud L., Timmis K.N. (2010). Cold-Adapted hydrocarbon-Degrading microorganisms. Handbook of Hydrocarbon and Lipid Microbiology.

[9] Crisafi F., Giuliano L., Yakimov M.M., Azzaro M., Denaro R. (2016). Isolation and degradation potential of a cold-adapted oil/PAHdegrading marine bacterial consortium from Kongsfjorden (*Arctic region*). Rend. Lincei Sci. Fis. E Nat..

[10] Yakimov M.M., Giuliano L., Gentile G., Crisafi E., Chernikova T.N., Abraham W.R., Lunsdorf H., Timmis K.N., Golyshin P.N. (2003). *Oleispira antarctica* gen. nov., sp. nov., a novel hydrocarbonoclastic marine bacterium isolated from Antarctic coastal sea water. Int. J. Syst. Evol. Microbiol..

[11] Gentile G., Bonasera V., D’Amico C., Giuliano L., Yakimov M.M. (2003). *Shewanella* sp. GA-22, a psychrophilic hydrocarbonoclastic Antarctic bacterium producing polyunsaturated fatty acids. J. Appl. Microbiol..

[12] Michaud L., Lo Giudice A., Saitta M., De Domenico M., Bruni V. (2004). The biodegradation efficiency on diesel oil by two psychrotrophic Antarctic marine bacteria during a two-month-long experiment. Mar. Pollut. Bull..

[13] Yakimov M.M., Gentile G., Bruni V., Cappello S., D’Auria G., Golyshin P.N., Giuliano L. (2004). Crude oil-induced structural shift of coastal bacterial communities of Rod Bay (Terra Nova Bay, Ross Sea) and characterization of cultured cold-adapted hydro-carbonoclastic bacteria. FEMS Microbiol. Ecol..

[14] Pini F., Grossi C., Nereo S., Michaud L., Lo Giudice A., Bruni V., Baldi F., Fani R. (2007). Molecular and physiological characterisation of psychotrophic hydrocarbon-degrading bacteria isolated from Terra Nova Bay (Antarctica). Eur. J. Soil Biol..

[15] Röling W.F.M., Milner M.G., Jones D.M., Lee K., Daniel F., Swannell R.P.J., Head I.M. (2002). Robust hydrocarbon degradation during nutrient-enhanced oil spill bioremediation. Appl. Environ. Microbiol..

[16] Das N., Chandran P. (2011). Microbial degradation of petroleum hydrocarbon contaminants: An overview. Biotechnol. Res. Int..

[17] Bell T.H., Yergeau E., Maynard C., Juck D., Whyte L.G., Greer C.W. (2013). Predictable bacterial composition and hydrocarbon degradation in Arctic soils following diesel and nutrient disturbance. ISME J..

[18] Garneau M., Michel C., Meisterhans G., Fortin N., King T., Greer C., Lee K. (2016). Hydrocarbon biodegradation by Arctic sea-ice and sub-ice microbial communities during microcosm experiments, Northwest Passage (Nunavut, Canada). FEMS Microbiol. Ecol..

[19] Amalfitano S., Fazi S. (2008). Recovery and quantification of bacterial cells associated with streambed sediments. J. Microbiol. Methods.

[20] Amalfitano S., Fazi S., Ejarque E., Freixa A., Romaní A.M., Butturini A. (2018). Deconvolution model to resolve cytometric microbial community patterns in flowing waters. Cytom. Part A.

[21] Luna G.M., Dell’Anno A., Danovaro R. (2006). DNA extraction procedure: A critical issue for bacterial diversity assessment in marine sediments. Environ. Microbiol..

[22] Lukow T., Dunfield P.F., Liesack W. (2000). Use of the T-RFLP technique to assess spatial and temporal changes in the bacterial community structure within an agricultural soil planted with transgenic and non-transgenic potato plants. FEMS Microbiol. Ecol..

[23] Smith C.J., Danilowicz B.S., Clear A.K., Costello F.J., Wilson B., Meijer W.G.T. (2005). Align, a web-based tool for comparison of multiple terminal restriction fragment length polymorphism profiles. FEMS Microbiol. Ecol..

[24] Baldi F., Marchetto D., Pini F., Fani R., Michaud L., Lo Giudice A., Berto D., Giani M. (2010). Biochemical and microbial features of shallow marine sediments along the Terra Nova Bay (Ross Sea, Antarctica). Cont. Shelf Res..

[25] Engebretson J.J., Moyer C.L. (2003). Fidelity of select restriction endonucleases in determining microbial diversity by terminal-restriction fragment length polymorphism. Appl. Environ. Microbiol..

[26] Danovaro R., Luna G.M., Dell’Anno A., Pietrangeli B. (2006). Comparison of two fingerprinting techniques, terminal restriction fragment length polymorphism and automated ribosomal intergenic spacer analysis, for determination of bacterial diversity in aquatic environments. Appl. Environ. Microbiol..

[27] Gerçe B., Schwartz T., Syldatk C., Hausmann R. (2011). Differences between bacterial communities associated with the surface or tissue of Mediterranean sponge species. Microb. Ecol..

[28] Altschul S.F., Gish W., Miller W., Myers E.W., Lipman D.J. (1990). Basic local alignment search tool. J. Mol. Biolology.

[29] Mason O.U., Hazen T.C., Borglin S., Chain P.S.G., Dubinsky E.A., Fortney J.L., Han J., Holman H.Y., Hultman J., Lamendella R. (2012). Metagenomics, metatranscriptomics and single cell sequencing reveal bacterial response to the Gulf oil spill. ISME J..

[30] Yergeau E., Maynard C., Sanschagrin S., Champagne J., Juck D., Lee K., Greer C.W. (2015). Microbial community composition, functions, and activities in the Gulf of Mexico 1 year after the Deepwater Horizon accident. Appl. Environ. Microbiol..

[31] Bacosa H.P., Erdner D.L., Rosenheim B.E., Shetty P., Seitz K.W., Baker B.J., Liu Z. (2018). Hydrocarbon degradation and response of seafloor sediment bacterial community in the northern Gulf of Mexico to light Louisiana sweet crude oil. ISME J..

[32] Acosta-González A., Martirani-von Abercron S.M., Rosselló-Móra R., Wittich R.M., Marqués S. (2015). The effect of oil spills on the bacterial diversity and catabolic function in coastal sediments: A case study on the Prestige oil spill. Environ. Sci. Pollut. Res..

[33] Duran R., Cravo-Laureau C. (2016). Role of environmental factors and microorganisms in determining the fate of polycyclic aromatic hydrocarbons in the marine environment. FEMS Microbiol. Rev..

[34] Yang T., Nigro L.M., Gutierrez T., D’Ambrosio L., Joye S.B., Highsmith R., Teske A. (2016). Pulsed blooms and persistent oil-degrading bacterial populations in the water column during and after the Deepwater Horizon blowout. Deep Sea Res. Part II Top. Stud. Oceanogr..

[35] Saul D.J., Aislabie J.M., Brown C.E., Harris L., Foght J.M. (2005). Hydrocarbon contamination changes the bacterial diversity of soil from around Scott Base, Antarctica. FEMS Microbiol. Ecol..

[36] Horz H.P., Tchawa Yimga M., Liesack W. (2001). Detection of methanotroph diversity on roots of submerged rice plants by molecular retrieval of *pmoA, mmoX, mxaF*, and 16S rRNA and ribosomal DNA, including terminal restriction fragment length polymorphism profiling. Appl. Environ. Microbiol..

[37] Sipos R., Székely A.J., Palatinszky M., Révész S., Márialigeti K., Nikolausz M. (2007). Effect of primer mismatch, annealing temperature and PCR cycle number on 16S rRNA gene-targeting bacterial community analysis. FEMS Microbiol. Ecol..

[38] Tiquia S.M., Michel F.C., Insam H. (2002). Bacterial diversity in livestock manure composts as characterized by terminal restriction fragment length polymorphisms (T-RFLP) of PCR-amplified 16S rRNA and gene sequences. Microbiology of Composting and Other Biodegradation Processes.

[39] Sanchez J.I., Rossetti L., Martinez B., Rodriguez A., Giraffa G. (2006). Application of reverse transcriptase PCR-based T-RFLP to perform semi-quantitative analysis of metabolically active bacteria in dairy fermentations. J. Microbiol. Methods.

[40] Moeseneder M.M., Arrieta J.M., Muyzer G., Winter C., Herndl G.J. (1999). Optimization of terminal-restriction fragment length polymorphism analysis for complex marine bacterioplankton communities and comparison with denaturing gradient gel electrophoresis. Appl. Environ. Microbiol..

[41] Nikolausz M., Sipos R., Revesz S., Szekely A., Marialigeti K. (2005). Observation of bias associated with re-amplification of DNA isolated from denaturing gradient gels. FEMS Microbiol. Lett..

[42] Nunan N., Daniell T.J., Singh B.K., Papert A., McNicol J.W., Prosser J.I. (2005). Links between plant and rhizoplane bacterial communities in grassland soils, characterized using molecular techniques. Appl. Environ. Microbiol..

[43] Eriksson M., Ka J.O., Mohn W.W. (2001). Effects of low temperature and freeze-thaw cycles on hydrocarbon biodegradation in arctic tundra soil. Appl. Environ. Microbiol..

[44] Whyte L.G., Smits T.H.M., Labbè D., Witholt B., Greer C.W., Van Beilen J.B. (2002). Gene cloning and characterization of multiple alkane hydroxilase systems in *Rhodococcus* strains Q15 and NRRL B*-*16531. Appl. Environ. Microbiol..

[45] Yakimov M.M., Timmis K.N., Golyshin P.N. (2007). Obligate oil-degrading marine bacteria. Curr. Opin. Biotechnol..

[46] Kube M., Chernikova T.N., Al-Ramahi Y., Beloqui A., Lopez-Cortez N., Guazzaroni M.-A., Heipieper H.J., Klages S., Kotsyurbenko O.R., Langer I. (2013). Genome sequence and functional genomic analysis of the oil-degrading bacterium *Oleispira antarctica*. Nat. Commun..

[47] Coulon F., McKew B.A., Osborn A.M., McGenity T.J., Timmis K.N. (2007). Effects of temperature and biostimulation on oil-degrading microbial communities in temperate estuarine waters. Environ. Microbiol..

[48] Gentile G., Bonsignore M., Santisi S., Catalfamo M., Giuliano L., Genovese L., Yakimov M.M., Denaro R., Genovese M., Cappello S. (2016). Biodegradation potentiality of psychrophilic bacterial strain *Oleispira antarctica* RB-8T. Mar. Pollut. Bull..

[49] Whyte L.G., Hawari J., Zhou E., Bourbonniére L., Inniss W.E., Greer C.W. (1998). Biodegradation of variable chain-length alkanes at low temperatures by a psychrotrophic *Rhodococcus* sp.. Appl. Environ. Microbiol..

[50] Grossman M.J., Prince R.C., Garrett R.M., Garrett K.K., Bare R.E., O’Neil K.R., Sowlay M.R., Hinton S.M., Lee K., Sergy G., Bell C.R., Brylinsky M., Johnson-Green P. (2000). Microbial diversity in oiled and un-oiled shoreline sediments in the Norwegian Arctic. Microbial Biosystems: New Frontiers Proceedings of the 8th International Symposium on Microbial Ecology.

[51] Terrisse F., Cravo-Laureau C., Noël C., Cagnon C., Dumbrell A.J., McGenity T.J., Duran R. (2017). Variation of oxygenation conditions on a hydrocarbonoclastic microbial community reveals *Alcanivorax* and *Cycloclasticus* ecotypes. Front. Microbiol..

[52] Brito E.M.S., Guyoneaud R., Goñi-Urriza M., Ranchou-Peyruse A., Verbaere A., Crapez M.A.C., Wasserman J.C.A., Duran R. (2006). Characterization of hydrocarbonoclastic bacterial communities from mangrove sediments in Guanabara Bay, Brazil. Res. Microbiol..

[53] LaRoe S.L., Wang B., Han J.I. (2010). Isolation and characterization of a novel polycyclic aromatic hydrocarbon-degrading bacterium, *Sphingopyxis* sp strain M2R2, capable of passive spreading motility through soil. Environ. Eng. Sci..

[54] Yergeau E., Sanschagrin S., Beaumier D., Greer C.W. (2012). Metagenomic analysis of the bioremediation of diesel- contaminated Canadian high arctic soils. PLoS ONE.

[55] Hwang C.Y., Lee I., Cho Y., Lee Y.M., Jung Y.J., Baek K., Nam S., Lee H.K. (2015). *Sediminicola arcticus* sp. nov., a psychrophilic bacterium isolated from deep-sea sediment, and emended description of the genus *Sediminicola*. Int. J. Syst. Evol. Microbiol..

