# Supplementary material for: Effects of a Simulated Acute Oil Spillage on Bacterial Communities from Arctic and Antarctic Marine Sediments
Source: Microorganisms. 2019 Nov 30;7(12):632. doi: 10.3390/microorganisms7120632 (PMC6956123; doi:10.3390/microorganisms7120632)
Supplement: Supplementary file 1 [file microorganisms-07-00632-s001.pdf]

Supplementary Materials

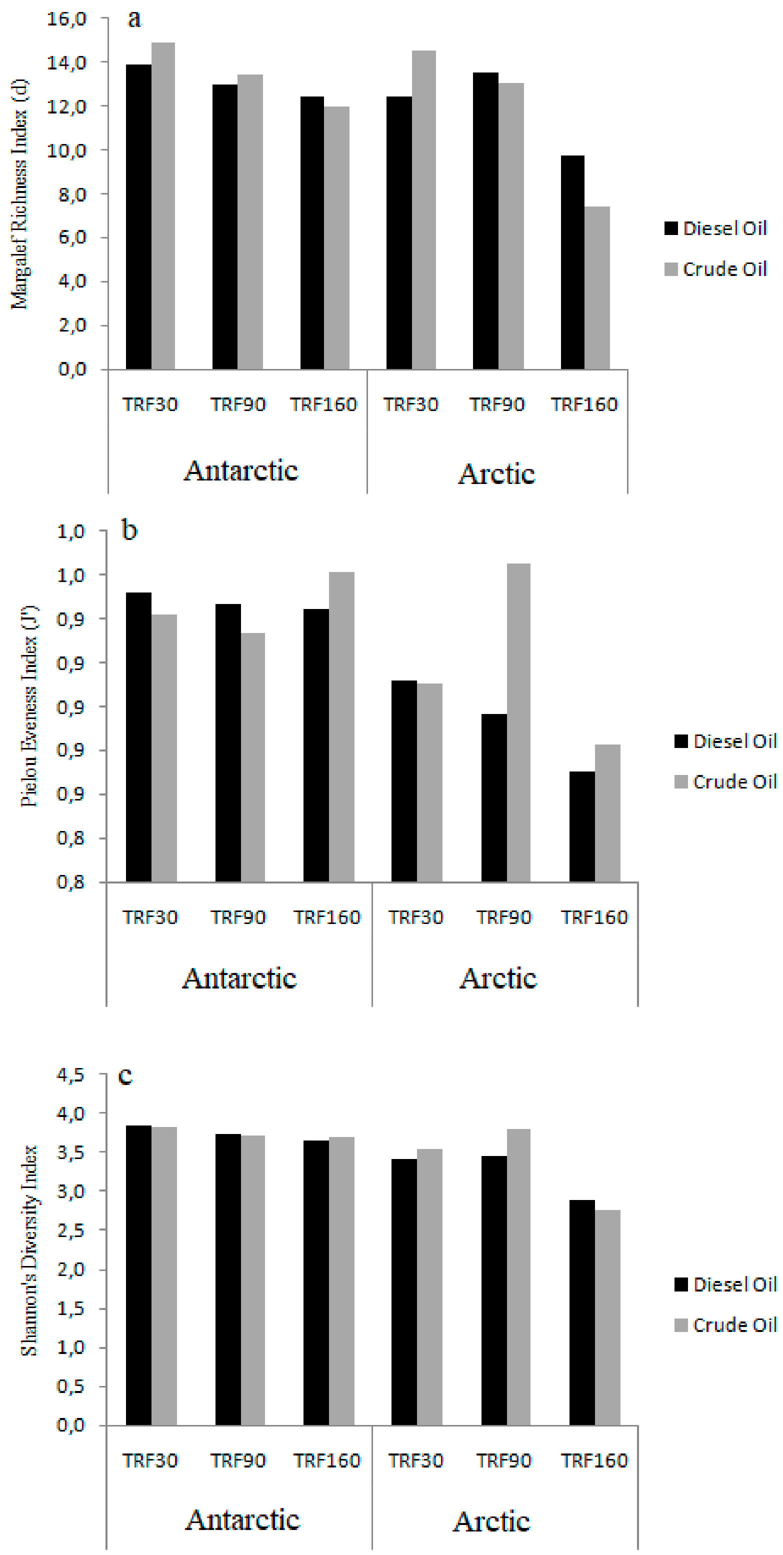

Figure 1. Richness, diversity and evenness indexes calculated from the T-RFLP data obtained from Arctic and Antarctic microcosms.

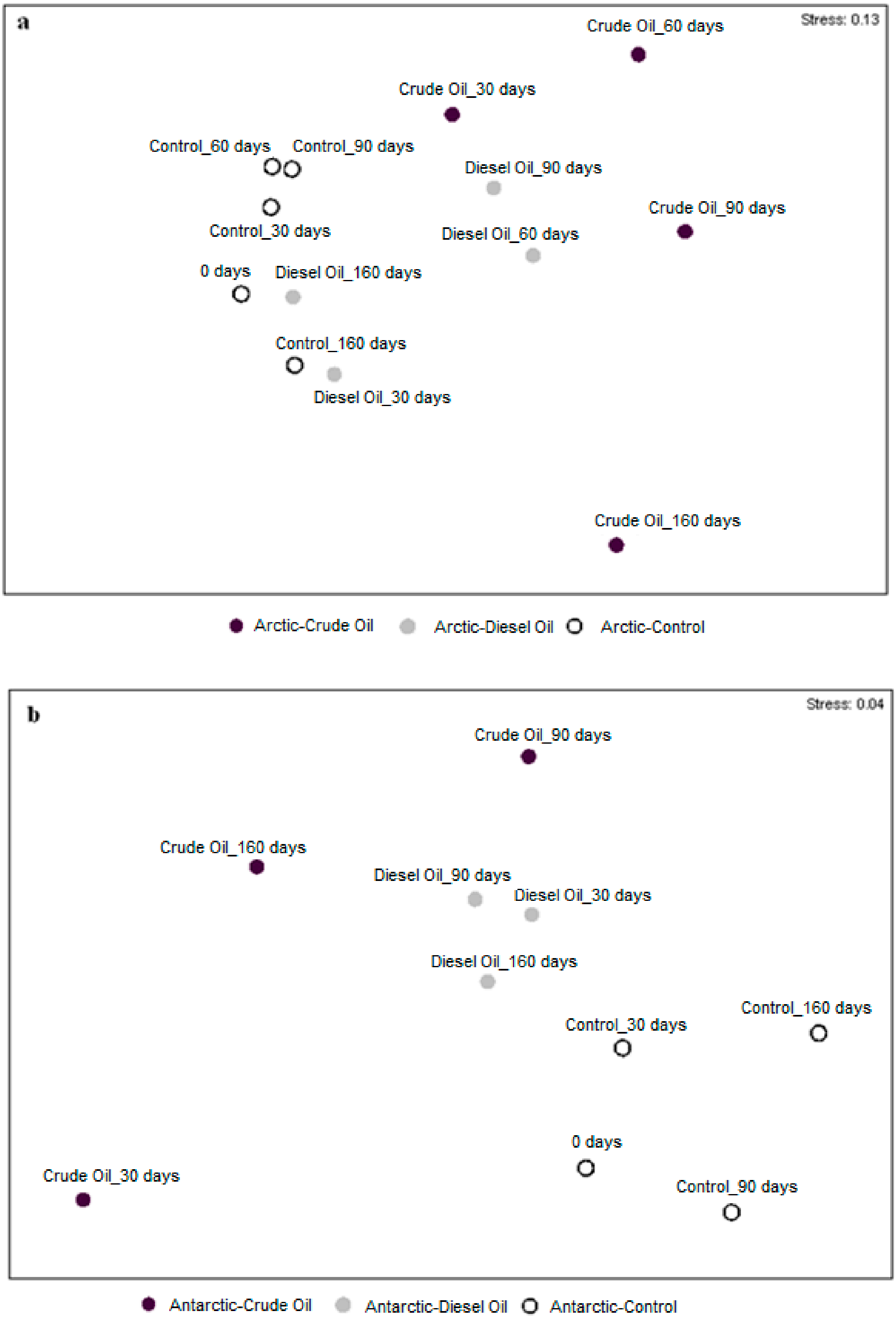

Figure 2. Non-metric multidimensional scaling (nMDS) computed by Bray-Curtis similarities calculated on T-RFLP results obtained from a) Arctic and b) Antarctic microcosms.

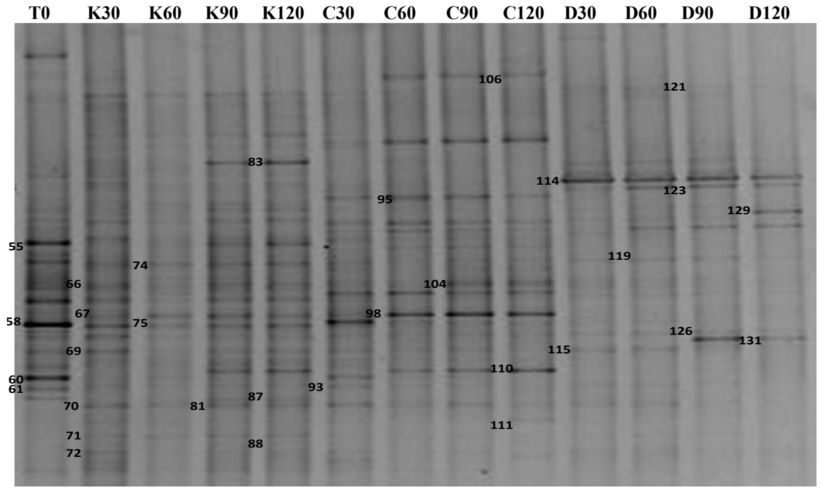

Figure 3. DGGE profiles of the representative 16S rDNA fragments obtained from Arctic sediment bacterial communities at different time of incubation (30, 60, 90 or 160 days). K, control; C, crude oil; D, diesel oil. The Arabic numbers indicate the DGGE bands that were sequenced.

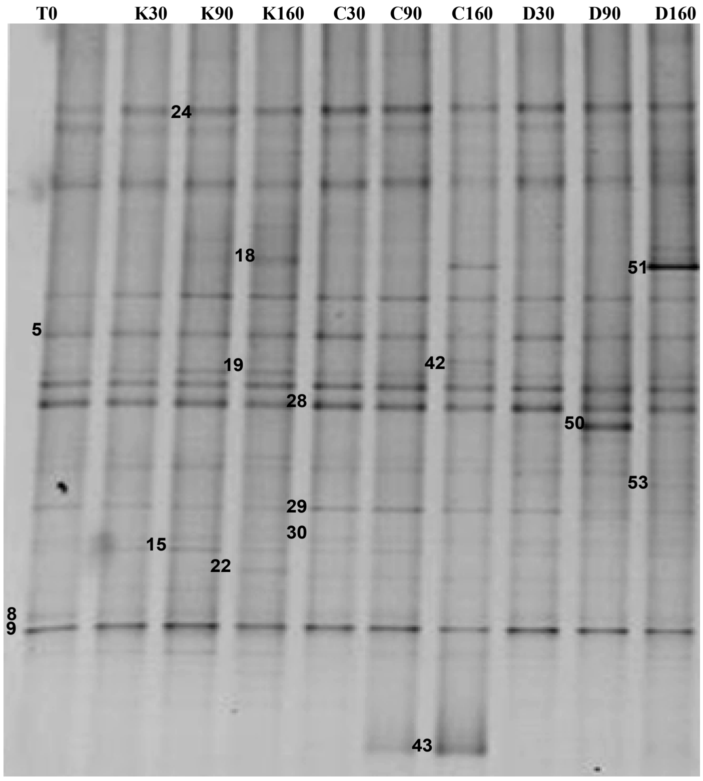

Figure 4. DGGE profiles of the representative 16S rDNA fragments obtained from Antarctic sediment bacterial communities at different incubation time (30, 90 or 160 days). K, control; C, crude oil; D, diesel oil. The Arabic numbers indicate the DGGE bands that were sequenced.
